# Pediatric extracorporeal cardiopulmonary resuscitation: single-center study

**DOI:** 10.3906/sag-2002-10

**Published:** 2021-08-30

**Authors:** Tanıl KENDİRLİ, Serhan ÖZCAN, Merve HAVAN, Cağdaş BARAN, Mehmet ÇAKICI, Burcu ARICI, Özlem SELVİ CAN, Zeynep EYİLETEN, Tayfun UÇAR, Ercan TUTAR, Ahmet Rüçhan AKAR

**Affiliations:** 1 Department of Pediatric Critical care Medicine, Faculty of Medicine, Ankara University, Ankara Turkey; 2 Department of Cardiovascular Surgery, Faculty of Medicine, Ankara University, Ankara Turkey; 3 Department of Anesthesiology and Reanimation, Faculty of Medicine, Ankara University, Ankara Turkey; 4 Department of Pediatric Cardiology, Faculty of Medicine, Ankara University, Ankara Turkey

**Keywords:** Extracorporeal cardiopulmonary resuscitation, extracorporeal membrane oxygenation, children

## Abstract

**Background/aim:**

Extracorporeal cardiopulmonary resuscitation (ECPR) is defined as the venoarterial extracorporeal membrane oxygenation (VA-ECMO) support in a patient who experienced a sudden pulseless condition attributable to cessation of cardiac mechanical activity and circulation. We aimed to evaluate the clinical outcomes of our ECPR experience in a pediatric patient population.

**Materials and methods:**

Between September 2014 and November 2017, 15 children were supported with ECPR following in-hospital cardiac arrest (IHCA) in our hospitals. VA-ECMO setting was established for all patients. Pediatric cerebral performance category (PCPC) scales and long-term neurological prognosis of the survivors were assessed.

**Results:**

The median age of the study population was 60 (4–156) months. The median weight was 18 (4.8–145) kg, height was 115 (63–172) cm, and body surface area was 0.73 (0.27–2.49) m^2^. The cause of cardiac arrest was a cardiac and circulatory failure in 12 patients (80%) and noncardiac causes in 20%. Dysrhythmia was present in 46%, septic shock in 13%, bleeding in 6%, low cardiac output syndrome in 13%, and airway disease in 6% of the study population. Median low-flow time was 95 (range 20–320) min. Central VA-ECMO cannulation was placed in only 2 (13.3%) cases. However, the return of spontaneous circulation (ROSC) was obtained in 10 (66.6%) patients, and 5 (50%) of them survived. Overall, 5 patients were discharged from the hospital. Finally, survival following ECPR was 33.3%, and all survivors were neurologically intact at hospital-discharge.

**Conclusion:**

ECPR can be a life-saving therapeutic strategy using a promising technology in the pediatric IHCA population. Early initiation and a well-coordinated, skilled, and dedicated ECMO team are the mainstay for better survival rates.

## 1. Introduction

Emergency venoarterial extracorporeal membrane oxygenation (VA-ECMO) in the setting of cardiac arrest refractory to conventional measures is defined as extracorporeal cardiopulmonary resuscitation (ECPR) [1]. Over the last decade, there has been a 10-fold increase in ECPR statistics in the Extracorporeal Life Support Organization (ELSO) registry^1^Extracorporeal Life Support Organization. ELSO Guidelines for ECPR Cases. Version 1.3 [online]. Website http://www.elso.org/Portals/0/IGD/Archive/FileManager/6713186745cusersshyerdocumentselsoguidelinesforecprcases1.3.pdf. [accessed 30, March, 2020.)] [1]. However, in the current advanced pediatric life support guidelines, the employment of ECPR is still only reluctantly recommended and restricted to children with cardiac disease at specialized centers, mainly due to limited evidence concerning the improvement of survival and functional outcome by ECPR in addition to a significant knowledge gap [1,2]. According to ELSO, ECPR is denoted as the implementation of extracorporeal life support (ECLS) as part of the resuscitation in cardiac arrest. Hemodynamically unstable patients placed on ECLS emergently without a cardiac arrest are not considered as ECPR^2^International Summary of ELSO Registry Report, 2020 January, p. 1-2. https://www.elso.org/Registry/Statistics/InternationalSummary.aspx (accessed 15, April, 2020), ECMO support in the setting of active ECPR is challenging in both pediatric and adult populations. However, the field is advancing and more robust recommendations are given in all ages, especially for in-hospital cardiac arrest (IHCA) and primary cardiac disease. In the last decades, ECPR aimed to encourage reversibility of life-threatening events and anticipation of normal or near-normal neurological and physical functions [2].

Current data from the ELSO international registry of neonatal and pediatric ECPR show an overall survival to hospital discharge of approximately 42% [1]^2^. According to the 2020 ELSO report, ECMO support was provided to 129,037 patients, and 27,829 (21.5%) of them were children. ECPR was performed to 15,055 (11.6%) patients in all neonatal, pediatric, and adult age groups. Survival rates were 42%, 42%, and 29% in neonatal, pediatric, and adult age groups, respectively^2^.

ECPR requires significant medical and financial resources, and it may also cause substantial morbidity. Therefore, before the cannulation, the prediction of the patient’s survival probability is essential. Unfortunately, there is not yet enough clinical and laboratory data to identify the appropriate pediatric candidates for ECPR. Besides, the pediatric ECPR technique is more complicated than adult ECPR and it requires tools and instruments in various sizes, and an experienced team. There are quite a few pediatric ECPR reports from Turkey [5,6]. Here, we summarize our experience in performing pediatric ECPR.

## 2. Materials and methods

### 2.1. Research objective and inclusion criteria

This study was conducted in a 15-bed tertiary-care pediatric intensive care unit (PICU) at the Ankara University Faculty of Medicine, Cardiovascular surgery and perfusion services are readily available for consultation in our hospital on a 7/24-h basis. The Ethics Committee of Ankara University approved this study.

We retrospectively reviewed the medical records of the pediatric (age <18 years) ECPR-supported patients from September 2014 to November 2017. Inclusion criteria are any set-up of ECMO during ongoing CPR. Patients electively placed on VA-ECMO or transitioned from cardiopulmonary bypass to VA-ECMO in the operating room were excluded. 

### 2.2. Methodology

Recorded demographic and clinical data were evaluated. Demographic data included each patient’s age, sex, and underlying diagnosis. Clinical data included CPR duration, ECMO duration, ECMO cannulation, and survival. Indications for pediatric ECPR are based on the following criteria: (1) witnessed arrest; (2) rapid institution of CPR with medications and external cardiac massage; (3) no recovery of cardiac function within 10–20 min of the initiation of CPR; (4) no contraindications for mechanical support including the preexisting severe neurologic deficit, or multiorgan failure [5,6,]. A severe congenital abnormality, intracranial hemorrhage, or coagulopathy were contraindications for ECPR. Our institutional experiences of cannulation during ECPR is the neck region (internal jugular vein and carotid artery) for infants weighing less than 15 kg, and the femoral area (femoral vein and femoral artery) for children weighing more than 15 kg. Central cannulation was preferred when cardiac arrest developed after separation from cardiopulmonary bypass in the operating room during open-heart surgery.

**Figure 1:**
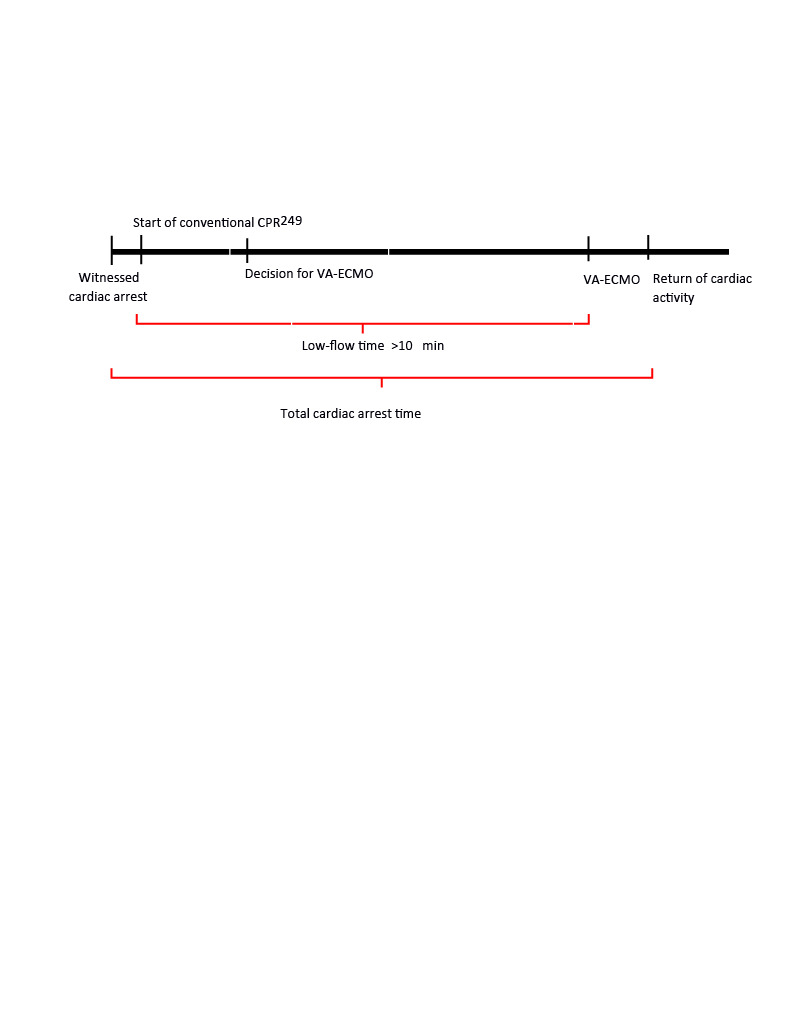
CPR-ECPR duration diagram showing low-flow and total cardiac arrest times. ECPR, extracorporeal cardiopulmonary resuscitation; CA, cardiac arrest; CPR, cardiopulmonary resuscitation; VA-ECMO, venoarterial extracorporeal membrane oxygenation.

Low-flow time was defined as the time interval from the start of conventional CPR (chest compressions or open cardiac massage) to the initiation of VA-ECMO support. Total cardiac arrest time was defined as the time interval between the onset of witnessed cardiac arrest and the return of cardiac activity or ECPR (Figure 1). VA-ECMO time was defined as the time from the start of VA-ECMO to the decannulation procedure. 

Neurologic outcome was evaluated using the pediatric cerebral performance category (PCPC) scale, grading functional neurologic status and PCPC scale include 1 for good, 2 for mild disability, 3 for moderate disability, 4 for severe disability, and 5 for vegetative state or coma (6 indicates death, but was not included in the study). A PCPC value of ≤ 2 is accepted as a good neurologic outcome [7]. 

### 2.3. Statistical analysis

All data were analyzed using SPSS v.20 (IBM, Armonk, NY, USA) software. We determined numerical values in categorical groups. Quantitative data results were expressed as median (minimum–maximum) in unsteady and average ± standard deviation in steady values. 

## 3. Results

**Figure 2:**
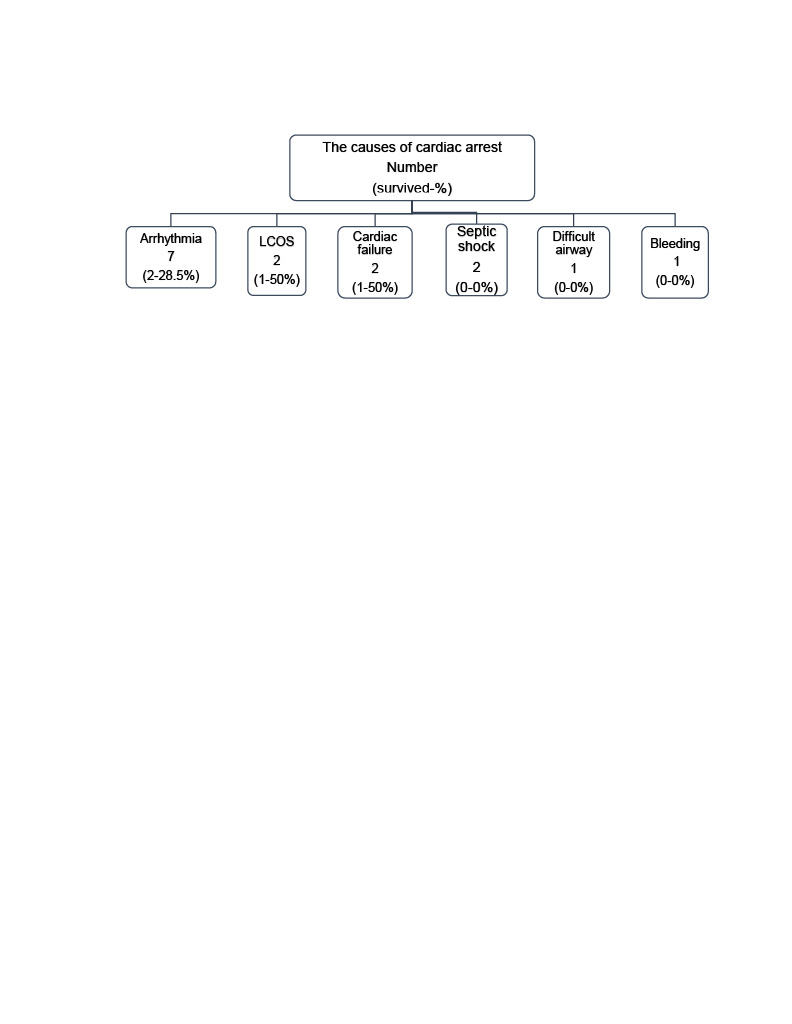
The causes of cardiac arrest. LCOS = low cardiac output syndrome.

In the study period, 15 children (8 female, 7 male) underwent ECPR in our hospital. Their median age, weight, height, and body surface area were 60 (4–156) months, 18 (4.8–145) kg, 115 (63–172) cm, and 0.73 (0.27–2.49) m^2^ respectively. They were all in-hospital cardiac arrests. Their primary etiology for cardiac arrest was a cardiac and circulatory failure in 80% of patients, and noncardiac causes in 20% (Table). The origins of cardiac arrest were dysrhythmia (ventricular fibrillation and tachycardia) in 46%, septic shock in 13%, bleeding in 6%, low cardiac output syndrome after congenital cardiac surgery in 13%, and airway disease in 6% of patients (Figure 2). All of the patients were supported with mechanical ventilation, and five of them needed continuous renal replacement treatment (CRRT) during VA-ECMO support. Post-CPR acute kidney injury was present in all patients; oliguria/anuria was detected in 3 patients. 

**Table:** Patients’ demographic information, and the features of extracorporeal cardiopulmonary resuscitation.

Patients	F/M	Age (months)	Weight(kg)	Primary diagnosesOperation/procedure	Procedure	The cause of cardiac arrest	Place of cannulation	The site of cannulas	Low-flow time (min)	Survived
1	F	144	40	Type 1 hyperlipidemiaAortic valve stenosis	AVR	Arrhythmia	PICU	FV-FA	70	Yes
2	F	132	145	S-CMP	None	Sepsis	PICU	Not able to be placed	145	No
3	M	60	20	RCMP,	Heart Tx	Bleeding	PICU	FV-FA	100	No
4	F	96	18	DCMP,	Heart Tx	Arrhythmia	PICU	FV-FA	20	Yes
5	F	4	4.8	AVSD	TC	LCOS	OR	Central	45	No
6	M	8	6	TAPVR, ASD, dextrocardia	None	Hypoxemia	OR	IJV-CA	110	No
7	F	42	12	Hydroxyflouric acid poisoning	None	Malignant arrhythmias	PICU	FV-FA	120	No
8	M	156	40	Lighter gas inhalation(poisoning)	None	Malignant arrhythmias	PICU	Not able to placed	105	No
9	M	24	10	ARDS, septic shock	None	Sepsis	OR	Not able tobe placed	120	No
10	F	23	12	Fulminant myocarditis	None	Cardiac failure	OR	IJV- CA	320	No
11	M	156	30	Danon disease	None	Arrhythmia	PICU	FV-FA	45	No
12	M	156	54	DCMP	Heart Tx	Cardiac failure	PICU	FV-FA	60	Yes
13	M	132	22	ARVD/C	None	Arrhythmia	OR	FV-FA	45	Yes
14	F	12	7	CoA, PDA ligation	TC	Arrhythmia	OR	IJV-CA	90	No
15	F	5	6	ASD and VSD	TC	LCOS	OR	Central	120	Yes

F/M, female/male; ECPR, extracorporeal membrane oxygenation; PC, postcardiotomy; S-CMP, sepsis-related cardiomyopathy; RCMP, restrictive cardiomyopathy; DCMP, dilated cardiomyopathy; AVR, aortic valve replacement; FV, femoral vein; FA, femoral artery; M, male; F, female; OR, operating room; TAPVR, total anomalous pulmonary venous return; AVSD, atrioventricular septal defect; PDA, patent ductus arteriosus; ASD, atrial septal defect; VSD, ventricular septal defect; TC, total correction; ARVD/C, arrhythmogenic right ventricular dysplasia/cardiomyopathy; CoA, coarctation of aorta; LCOS, low cardiac output state; IJV, internal jugular vein; CA, carotid artery; heart Tx, heart transplantation.

ECPR was performed in the PICU in 8 patients and 7 patients in the operation room. Median low-flow time was 95 (ranged 20–320) min. ECMO cannulas were placed in the femoral vein-femoral artery in 7 patients, and internal jugular vein-carotid artery in 3 patients. Central cannulation was established in 2 patients. We could not obtain sufficient blood flow despite successful cannulation in 2 patients (one central cannulation and one femoral vein-femoral artery cannulation). It was not possible to place cannulas in 3 patients; hence, they were excluded from the study.

We also had the first pediatric ECMO transport experience in Turkey. The child (no. 10) suffered from acute fulminant myocarditis. Our ECMO team went to Erzurum, 800 km away from our center to set up ECMO. After she had undergone active CPR for about 4 h, ECMO was set up and then transferred to our PICU by an airplane ambulance. Thus, she had the most extended ECPR duration in our study, with 320 min (Table).

ECMO was established in 10 (66.6%) patients successfully, and their mean ECMO flow rate was 1950 ± 1300 mL/m^2^/min. The range of the VA-ECMO time after ECPR was between 12 h and 18 days. Of these 10 patients, 5 survived to hospital discharge as survival was 33.3%. All of them have favorable neurologic outcomes (PCPC ≤ 2). 

## 4. Discussion

The reported survival rates after pediatric cardiac arrest are 9% to 47% for IHCA [10]. Achieving acceptable survival rates in the pediatric population is a challenging task that requires a multidisciplinary dedicated team available for 24-h. The reported survival rate after pediatric ECPR is 23%–51% [1,8–10], which is better than conventional CPR. Despite the different survival rates among a variety of patient groups, these reports indicate that ECPR may have a favorable outcome for pediatric cardiac arrests. In this study, we have tried to apply ECPR to 15 children. We succeeded in 10 patients (66.6%). Among ten, 4 died because of the extensive bleeding due to disseminated intravascular coagulation. One patient (number 10) who had acute fulminant myocarditis was transported with VA-ECMO support in an airplane ambulance after 4 h of CPR. Subsequently, she had a brain death in PICU.

Reports of outcomes regarding survival to hospital discharge are also variable. Kelly RB et al. [8] analyzed 31 pediatric patients who received ECPR. In their study, 7 (23%) patients survived to hospital discharge, and 24 patients died. In our study, ECPR successfully rescued 5 patients who ultimately survived and were discharged from the hospital. Moreover, they had favorable neurologic outcomes at the time of hospital discharge.

ECPR is a potentially life-saving intervention, but it carries significant risks. Therefore, it is imperative to decide which pediatric patients would benefit from ECPR. Witnessed arrest and rapid application of CPR are essential for the indication of ECPR [5,6]. We decided to ECPR if there was no recovery of cardiac function within 10–20 min of the conventional CPR in the selected patients. ECPR decision time was not available since this is a retrospective study. 

The median CPR duration in children is 46–60 min according to different reports [8,10]. In our study, the median CPR duration was longer at 78 min. Prolonged CPR duration decreases the survival rate in adults [8,9]. High-quality CPR is the most crucial factor for favorable neurologic outcomes [8,10,11]. Therefore, reducing CPR duration and maintaining high-quality CPR may improve the survival rate and neurologic issues.

We are currently trying to shorten the low-flow interval in our unit by decreasing the time for preparation and cannulation. The ECMO team (pediatric intensivist, cardiovascular surgeon, and perfusionist) is available for deployment of ECPR on a 24-h basis. We provide a dedicated ECMO device, circuit, and cannulas in the PICU. All cannulations are performed by attending cardiovascular surgeons. Patient status determines the cannulation site. In this study, cannulation is generally performed peripherally (the carotid artery and jugular vein in infants and toddlers, and the femoral artery and vein in older children and adolescents). Two central cannulations were performed following cardiac surgery.

For successful clinical outcomes, it is essential to determine the indication criteria for ECPR. It is also vital to reduce the duration of ECMO preparation and the interval before the arrival of the cardiovascular surgeon. Therefore, team communication and synchronization are critical.

In conclusion, ECPR is a life-saving therapy, especially for children who have reversible causes of cardiac arrest and exposed to prolonged CPR. In the interest of good neurologic outcomes among the survivors, we suggest that all PICUs should be prepared for a pediatric ECPR at all times. 

## Author contributions

TK, ET, and ARA are joint principal authors who contributed to the conception and design of the study and conducted the data analysis and wrote the paper; MH and SÖ contributed equally to patients’ resuscitation and supported all treatments; TU, ZE, MÇ, and BA equally contributed to performing ECMO cannulation and monitoring the ECMO parameters.

## Data availability statement

The data that support the findings of this study are available on request from the corresponding author.

## Compliance with ethical standards 

All procedures performed in studies involving human participants were in accordance with the ethical standards of the institutional and/or national research committee and in accordance with the 1964 Helsinki declaration and its later amendments or comparable ethical standards.
